# Efficacy of conbercept pro re nata (PRN) versus treat-and-extend (T&E) regimens following five initial monthly doses in diabetic macular edema: a 24-month retrospective cohort study

**DOI:** 10.3389/fendo.2026.1875115

**Published:** 2026-08-26

**Authors:** Mengyao Yang, Pengfei Jiang, Yuanqing Liu, Jie Wang, Xiaonan Zhao, Nan Wang, Yuan Tao, Hong Wang

**Affiliations:** 1 Department of Ophthalmology, Qilu Hospital, Shandong University, Jinan, Shandong, China; 2 Department of Ophthalmology, Qingdao University, Affiliated Yantai Yuhuangding Hospital, Yantai, Shandong, China; 3 Jinan Huashi Ophthalmology Hospital, Shandong, China; 4 Jinan Aier Eye Hospital, Jinan, Shandong, China; 5 Department of Ophthalmology, Jinan Second People’s Hospital, Jinan, Shandong, China

**Keywords:** 5+PRN, 5+T&E, conbercept, diabetic macular edema, therapeutic effect

## Abstract

**Objective:**

This study aimed to observe and compare the visual and anatomical improvements over 24 months in patients with diabetic macular edema (DME) treated with intravitreal conbercept injection following five initial monthly doses, using either a Pro Re Nata (PRN) regimen or a Treat-and-Extend (T&E) regimen. The injection and visit frequency were also compared between the two groups.

**Methods:**

This study retrospectively analyzed 119 patients with DME. Sixty patients received the 5+PRN regimen, while the other 59 patients were treated with the 5+T&E regimen. Baseline and post-treatment parameters, including best-corrected visual acuity (BCVA), central foveal thickness (CFT), microaneurysm count (MA count), retinal vein diameter, deep capillary plexus vessel density (DCP-VD), foveal avascular zone (FAZ) area, hard exudate (HE) area, and hemorrhage area, were recorded and compared between the two groups. The total number of intravitreal injections administered within 24 months was documented for both groups.

**Results:**

Significant improvements in BCVA, CFT, and MA count were observed as early as 6 months after treatment in both groups, and these improvements were sustained through 24 months. Significant improvements in retinal vein diameter and DCP-VD became evident at 18 months post-treatment. Both the HE area and hemorrhage area showed significant improvements at 24 months after treatment. No statistically significant change in FAZ area was detected during the follow-up period. There were no significant differences in all therapeutic outcomes between the two groups. The mean number of injections within the 24-month follow-up was 14.02 ± 2.05 in the 5+PRN group and 11.44 ± 1.74 in the 5+T&E group. Meanwhile, the average number of follow-up visits was 24.89 ± 1.67 in the 5+PRN group and 13.94 ± 2.60 in the 5+T&E group. Both the injection frequency and follow-up visits were significantly lower in the 5+T&E group than in the 5+PRN group.

**Conclusion:**

Conbercept is safe and effective for the treatment of DME. Both the 5+PRN and 5+T&E regimens demonstrated significant improvements in visual acuity and retinal anatomical structure over the 24-month follow-up period. Over 24 months, the 5+T&E group had fewer mean injections and follow-up visits compared with the 5+PRN group, indicating that an individualized dosing strategy can reduce patient burden and healthcare costs while maintaining therapeutic efficacy.

## 1 Introduction

With rapid economic development, improved healthcare, and population aging, the life expectancy of diabetic patients has increased, leading to a corresponding increase in the prevalence of diabetic complications. According to the International Diabetes Federation (IDF), approximately 537 million adults had diabetes globally in 2021, a figure projected to reach 643 million by 2030 and 783 million by 2045 (1). As the duration of diabetes increases, the incidence of microvascular complications has increased substantially, particularly diabetic retinopathy (DR) and diabetic macular edema (DME). DR and DME have become the leading causes of vision loss and blindness in the working-age population. The global prevalence of vision-threatening DR and DME is estimated at 11.7% and 7.48%, respectively (2). Furthermore, the prevalence of DME is strongly correlated with the duration of diabetes; it is present in about 5% of patients with newly diagnosed type 2 diabetes and rises to approximately 30% after 20–30 years of disease (3). These trends establish DME as a significant and growing public health concern.

DME is characterized by the accumulation of intraretinal fluid (IRF) and/or subretinal fluid (SRF), resulting from an imbalance between fluid influx into and efflux out of the retina. Under physiological conditions, the integrity of the blood-retinal barrier (BRB), together with the active drainage functions of Müller and retinal pigment epithelium (RPE) cells, maintains fluid homeostasis, thereby preserving retinal dryness and optical clarity (4). The pathogenesis of DME is multifactorial and involves multiple molecular pathways. Under chronic hyperglycemia, inflammatory responses, oxidative stress, retinal hypoxia, and neurovascular unit (NVU) injury collectively disrupt the inner and outer BRB. These pathological alterations also compromise the normal function of Müller cells and RPE cells, resulting in impaired fluid clearance and subsequent intraretinal fluid accumulation, which ultimately leads to the development of DME (5).

Vascular Endothelial Growth Factor (VEGF) plays a central role in mediating BRB breakdown and driving neovascularization in DR and DME. Hyperglycemia, inflammation, and hypoxia trigger upregulation of VEGF expression in a variety of retinal cell types, including Müller cells, endothelial cells, pericytes, and RPE cells. The subsequent rise in VEGF levels exacerbates BRB dysfunction and aberrant angiogenesis, increasing vascular permeability and giving rise to the fluid accumulation that defines DME (6, 7).

Currently, anti-VEGF therapy is established as the first-line treatment for DME, and standardized treatment protocols are of paramount importance for patient outcomes. The primary anti-VEGF regimens used clinically include fixed-dosing (monthly or every 2 months) after initial loading doses, pro re nata (PRN) after loading doses, and treat-and-extend (T&E) after loading doses (8). Although effective, fixed dosing presents substantial challenges with respect to patient convenience and healthcare burden due to the need for frequent follow-up visits. Accordingly, PRN and T&E regimens have been more widely adopted in clinical settings owing to their greater flexibility.

The PRN regimen involves an initial loading phase of monthly anti-VEGF injections (typically 3 to 5 doses), followed by monthly monitoring visits where reinjection is administered only when predefined retreatment criteria are met. However, the requirement for monthly follow-up visits under the PRN protocol places a significant logistical burden on patients, which may impact compliance and treatment sustainability.

The T&E regimen begins with an initial phase of monthly anti-VEGF injections until disease activity is controlled. Thereafter, the treatment interval is individualized based on disease activity. At each visit, the patient receives an anti-VEGF injection, and the next appointment is scheduled. If no disease activity is observed, the treatment interval is extended (typically by 2 or 4 weeks); if signs of recurrence are present, the interval is shortened. The treatment interval generally ranges from a minimum of 4 weeks to a maximum of 12–16 weeks. Originally developed for the management of neovascular age-related macular degeneration (nAMD), the T&E regimen aims to reduce both the frequency of clinical visits and the number of injections, thereby reducing the burden on patients and the healthcare system. Likewise, a recent study on branch retinal vein occlusion (BRVO) indicated that the T&E regimen achieves improved efficacy while lowering the frequency of follow-ups and intravitreal injections (9).

In clinical practice, an early and intensive loading phase of anti-VEGF therapy is critical. This typically involves an initial series of 5–6 monthly injections. Following this, during the maintenance phase, the selection of a treatment regimen should be guided by a thorough assessment of disease activity and tailored to individual patient characteristics, with the goal of maximizing long-term treatment benefits.

Currently, clinical studies directly comparing the 5+PRN and 5+T&E regimens of conbercept in the treatment of DME remain scarce. This study was designed to evaluate and compare the 24-month visual acuity outcomes, anatomical improvements, and injection frequencies between these two treatment strategies, thereby providing new evidence to inform clinical decision-making in DME management.

## 2 Materials and methods

### 2.1 Patients

This single-center retrospective cohort study used data extracted from archived electronic medical records of routine clinical cases, and no patients were prospectively enrolled. A total of 119 patients with newly diagnosed DME treated at the Ophthalmology Clinic of Qilu Hospital, Shandong University between September 2020 and September 2022 were included. The diagnosis was confirmed by fundus examination, optical coherence tomography (OCT), OCT angiography (OCTA), and fundus fluorescein angiography (FFA). All included patients had regular follow-up records. Among them, 60 patients were divided into the 5+PRN regimen group, and 59 were divided into the 5+T&E regimen group. Written informed consent was obtained from all participants for retrospective analysis of their clinical data. The study was conducted in accordance with the principles of the Declaration of Helsinki, and full ethical approval was obtained from the Institutional Review Board of Qilu Hospital, Shandong University before data collection (KYLL-202209-048-2).

#### 2.1.1 Inclusion criteria

Inclusion criteria were as follows: (1) Patients diagnosed with type 2 diabetes with controlled glycemic status (HbA1c <10%) and blood pressure <160/90 mmHg; (2) Patients with presence of visual impairment or metamorphopsia; (3) DME confirmed by fundus examination, OCT, OCTA, and FFA, accompanied by a central foveal thickness (CFT) ≥ 300 μm; (4) Completion of regular follow-up for at least 24 months.

#### 2.1.2 Exclusion criteria

Exclusion criteria were as follows: (1) Patients diagnosed with diabetes types other than type 2; (2) Patients with ocular comorbidities such as cataract, vitreous hemorrhage, tractional retinal detachment, abnormal intraocular pressure, or any other condition causing significant media opacity or poor imaging quality; (3) Patients with previous ocular interventions, including panretinal photocoagulation, intravitreal injection of anti-VEGF agents or steroids, or vitrectomy; (4) Patients with coexisting retinal diseases such as age-related macular degeneration, retinal vein occlusion, choroidal neovascularization, or retinal detachment; (5) Patients with poor systemic health status; (6) Patients lost to follow-up after treatment initiation.

#### 2.1.3 Treatment groups

Eligible patients received an initial intravitreal injection of conbercept at the first visit, followed by monthly injections for a total of 5 doses. After the loading phase, patients were divided into two groups receiving the following treatment regimens based on their practical conditions and treatment preferences (e.g., geographical accessibility and feasibility of attending regular follow-up visits), rather than randomization.

5+PRN Group: After the 5 initial injections, patients were evaluated every 4 weeks. Reinjection was administered only if predefined retreatment criteria were met; otherwise, observation was continued.

5+T&E Group: After the 5 initial injections, the treatment interval was set to 8 weeks. At each subsequent visit, an injection was administered, and the next appointment was scheduled. If active disease was present, the interval was shortened (to a minimum of 4 weeks). If no disease activity was observed, the interval was extended by 4 weeks compared to the previous interval. This process was repeated at each visit, with the maximum interval not exceeding 16 weeks.

During the treatment period, rescue panretinal photocoagulation was allowed if neovascularization developed.

The retreatment criteria were defined as follows: (1) Presence of new or persistent IRF or SRF; (2) An increase in CFT of ≥ 50 μm compared to the previous measurement; (3) A loss of ≥ 5 letters in best-corrected visual acuity (BCVA) as measured on the Early Treatment Diabetic Retinopathy Study (ETDRS) chart.

All interventions were standard routine ophthalmic treatments.

### 2.2 Study methods

#### 2.2.1 Clinical baseline data collection

This single-center retrospective cohort study extracted data from archived electronic medical records of routine clinical cases with no prospective patient enrollment. Clinical information, including gender, age, affected eye, and duration of diabetes, was collected from electronic medical records.

#### 2.2.2 Ophthalmic examination

BCVA was measured using a standard ETDRS chart. Intraocular pressure (IOP) was obtained with a non-contact tonometer. Anterior segment evaluation was performed by slit-lamp biomicroscopy.

#### 2.2.3 Fundus photography

Color fundus images were acquired using a ZEISS VISUCAM 224 fundus camera. The same ophthalmologist manually delineated and measured the areas of all hard exudates and hemorrhages within a defined region of the posterior pole. This region was centered on the fovea, lay inside the superior and inferior vascular arcades, and extended from the optic disc margin to a point two disc diameters temporal to the fovea.

#### 2.2.4 Fundus fluorescein angiography

FFA images were acquired using the Heidelberg Spectralis HRA system. Venous diameter and microaneurysm counts were obtained based on FFA findings. Images centered on the optic disc and encompassing an area of 1 to 1.5 disc diameters were selected. The maximum projected diameters of the six largest veins were measured manually. The central retinal venular equivalent (CRVE) was then calculated using the revised Parr-Hubbard formula by Knudtson et al. and recorded as the venous diameter. The number of retinal microaneurysms within a 30-degree field centered on the fovea was manually counted at 1 minute after fluorescein injection.

#### 2.2.5 Optical coherence tomography

OCT images were acquired using the ZEISS Cirrus HD-OCT system. Linear scans were performed from superior to inferior across the macular region. A single cross-sectional image passing through the fovea center was selected, and the CFT was measured using the built-in image analysis software of the device. CFT was defined as the perpendicular distance from the inner limiting membrane to the inner boundary of the retinal pigment epithelium at the foveal center.

#### 2.2.6 Optical coherence tomography angiography

OCTA images were acquired using the ZEISS Cirrus HD-OCTA system. During image acquisition, the fovea was centered within a 6×6 mm square scanning area, generating concentric circles with diameters of 1, 3, and 6 mm centered on the fovea. The resulting images were saved for analysis. Vessel density in the deep capillary plexus (DCP-VD) and the foveal avascular zone (FAZ) parameters were analyzed using the proprietary software.

#### 2.2.7 Intravitreal injection

Patients were instructed to administer levofloxacin eye drops to the operative eye four times daily for three days before the procedure. One day before surgery, the lacrimal duct and conjunctival sac were irrigated to ensure ocular cleanliness. Topical anesthesia was administered 10 minutes before the procedure. An intravitreal injection of conbercept (0.05 mL) was administered in the superotemporal quadrant, 3.5 mm posterior to the corneal limbus. Following needle withdrawal, the injection site was compressed with a sterile cotton swab for 1 minute. After the patient was confirmed to have no significant discomfort, tobramycin-dexamethasone ophthalmic ointment was applied, and the eye was covered with a sterile eye patch.

#### 2.2.8 Panretinal photocoagulation

During the treatment period, rescue panretinal photocoagulation (PRP) was permitted if neovascularization developed. Nasally, the treatment boundary was positioned at ≥ 500 μm from the optic disc; temporally, at ≥ 3000 μm from the foveal center. The superior and inferior boundaries extended 1–3 spot diameters beyond the vascular arcades to the ocular equator. Laser parameters were set as follows: spot size of 200–500 μm, exposure duration of 0.1–0.3 seconds, and laser intensity sufficient to produce a mild gray-white retinal reaction (grade 2+ to 3+). Laser lesions were spaced 1–2 spot diameters apart. The entire treatment was completed in 2–4 sessions. All laser procedures were performed by the same senior vitreoretinal specialist.

#### 2.2.9 Clinical follow-up

At the 6-, 12-, 18-, and 24-month follow-up visits, BCVA, CFT, number of MAs, retinal venular diameter, DCP-VD, FAZ area, and the areas of HE and hemorrhage were recorded using the same methods described above. For patients who were unable to attend the scheduled visits, clinical data obtained within a 4-week window before or after each predefined time point were accepted as substitutes.

### 2.3 Statistical analysis

Statistical analysis was performed using SPSS version 25.0 (IBM Corporation, Armonk, NY, USA). Normally distributed quantitative data were presented as mean ± standard deviation. Comparisons between two groups were analyzed using the *t*-test, and multiple intergroup comparisons were conducted with the Student–Newman–Keuls (SNK) *q*-test. Categorical data were expressed as percentages (%), and intergroup differences were evaluated using the chi-square test. Statistical significance was established at *P* < 0.05.

## 3 Results

### 3.1 Baseline information

A total of 119 patients (119 eyes) were enrolled in this study, with 60 patients (60 eyes) assigned to the 5+PRN group and 59 patients (59 eyes) allocated to the 5+T&E group. No statistically significant differences were observed between the two groups in terms of baseline demographic characteristics, including gender, age, affected eye, and duration of diabetes. Furthermore, at baseline, there were no significant differences in BCVA, CFT, number of MAs, retinal venular diameter, DCP-VD, FAZ area, HE areas, and hemorrhage area (**Table 1**).

**Table 1:** Baseline information.

	5+PRN (n=60)	5+T&E (n=59)	*F*, *t*, *x^2^*	*P*
Sex,n (%)				
Male	28 (46.7%)	31 (52.5%)	0.5425	0.4614
Female	32 (53.3%)	27 (47.5%)		
Age (years)	62.53 ± 5.99	60.97 ± 6.61	1.3473	0.1805
Eyes,n (%)				
Right	35 (58.3%)	29 (49.2%)	1.0087	0.3152
Left	25 (41.7%)	30 (50.8%)		
Duration of diabetes (years)	6.35 ± 2.34	6.15 ± 2.13	0.4810	0.6314
BCVA (LogMAR)	0.68 ± 0.17	0.65 ± 0.17	1.2308	0.2209
CFT (μm)	498.14 ± 67.88	499.74 ± 77.45	0.1196	0.9050

BCVA, best-corrected visual acuity; CFT, central foveal thickness.

### 3.2 Changes in BCVA before and after treatment

At baseline, no statistically significant difference in BCVA (logMAR) was observed between the 5+PRN group (0.68 ± 0.17) and the 5+T&E group (0.65 ± 0.17). In the 5+PRN group, BCVA showed significant improvement at 6 months (0.49 ± 0.16), 12 months (0.49 ± 0.17), 18 months (0.47 ± 0.17), and 24 months (0.46 ± 0.18) after treatment compared with baseline (0.68 ± 0.17) (all *P* < 0.01). Similarly, in the 5+T&E group, BCVA was significantly improved at 6 months (0.46 ± 0.17), 12 months (0.50 ± 0.16), 18 months (0.52 ± 0.15), and 24 months (0.48 ± 0.18) after treatment compared with baseline (0.65 ± 0.17) (all *P* < 0.01). The greatest improvement in BCVA was observed at the 6-month follow-up in both groups, with the therapeutic effect remaining stable during the subsequent follow-up period. There were no significant differences in BCVA between the 5+PRN and 5+T&E groups at any post-treatment time point (6, 12, 18, or 24 months) (**Table 2**).

**Table 2:** Changes in BCVA before and after treatment.

BCVA	Baseline	6m	12m	18m	24m	*F*	*P*
5+PRN(n=60)	0.68 ± 0.17	0.49 ± 0.16	0.49 ± 0.17	0.47 ± 0.17	0.46 ± 0.18	18.09	0.0000
5+T&E(n=59)	0.65 ± 0.17	0.46 ± 0.17	0.50 ± 0.16	0.52 ± 0.15	0.48 ± 0.18	11.27	0.0000
*t*	1.2308	0.9565	0.0823	1.8450	0.5431		
*P*	0.2209	0.3408	0.9346	0.0676	0.5881		

In the 5+PRN group, compared with baseline, the *q*-values were 8.9371, 8.5386, 10.1751, and 9.9140 at 6, 12, 18, and 24 months after treatment, respectively (all *P* < 0.01). In the 5+T&E group, the corresponding *q*-values were 8.2793, 6.4683, 3.9040, and 7.5107 at each follow-up time point (all *P* < 0.01). Pairwise comparisons among the five sample means were conducted using the Newman–Keuls *q*-test.

BCVA, best-corrected visual acuity; m, months.

### 3.3 Changes in CFT before and after treatment

At baseline, there was no statistically significant difference in CFT between the 5+PRN group (498.14 ± 67.88 μm) and the 5+T&E group (499.74 ± 77.45 μm). Changes in CFT after treatment showed a trend generally consistent with that of BCVA. In both groups, the most pronounced reduction in CFT was observed at 6 months post-treatment, with a gradual decrease sustained throughout the subsequent treatment period. In the 5+PRN group, CFT decreased significantly at 6 months (341.13 ± 40.06 μm), 12 months (333.35 ± 47.69 μm), 18 months (335.43 ± 48.74 μm), and 24 months (325.13 ± 44.08 μm) compared with baseline (498.14 ± 67.88 μm) (all *P* < 0.01). Similarly, in the 5+T&E group, CFT was significantly reduced at 6 months (348.27 ± 31.58 μm), 12 months (342.41 ± 45.46 μm), 18 months (329.56 ± 44.88 μm), and 24 months (326.61 ± 45.33 μm) compared with baseline (499.74 ± 77.45 μm) (all *P* < 0.01). No statistically significant differences in CFT were found between the 5+PRN and 5+T&E groups at any of the post-treatment follow-up visits (6, 12, 18, or 24 months) (**Table 3**).

**Table 3:** Changes in CFT before and after treatment.

CFT(μm)	Baseline	6m	12m	18m	24m	*F*	*P*
5+PRN(n=60)	498.14 ± 67.88	341.13 ± 40.06	333.35 ± 47.69	335.43 ± 48.74	325.13 44.08	127.36	0.0000
5+T&E(n=59)	499.74 ± 77.45	348.27 ± 31.58	342.41 ± 45.46	329.56 ± 44.88	326.61 45.33	121.20	0.0000
*t*	0.1196	1.0781	1.0608	1.8450	0.1807			
*P*	0.9050	0.2832	0.2909	0.0676	0.8569			

In the 5+PRN group, compared with baseline, the *q*-values were 24.0301, 25.2211, 24.9031, and 26.4798 at 6, 12, 18, and 24 months after treatment, respectively (all *P* < 0.01). In the 5+T&E group, the corresponding *q*-values were 22.7025, 23.5802, 25.5071, and 25.9492 at each follow-up time point (all *P* < 0.01). Pairwise comparisons among the five sample means were conducted using the Newman–Keuls *q*-test.

CFT, central foveal thickness;m, months.

### 3.4 Changes in the number of MAs before and after treatment

At baseline, no statistically significant difference was observed in the number of MAs between the 5+PRN group (89.74 ± 22.10) and the 5+T&E group (84.29 ± 21.20). In the 5+PRN group, the number of MAs demonstrated a significant decrease at 6 months (59.63 ± 17.88), 12 months (44.56 ± 19.08), 18 months (48.32 ± 19.25), and 24 months (46.34 ± 17.51) after treatment compared to the baseline value (89.74 ± 22.10) (all *P* < 0.01). Similarly, a significant reduction in the number of MAs was noted in the 5+T&E group at 6 months (60.98 ± 20.83), 12 months (41.65 ± 17.73), 18 months (41.03 ± 17.87), and 24 months (43.52 ± 18.40) after treatment relative to baseline (84.29 ± 21.20) (all *P* < 0.01). The most pronounced reduction in MA count occurred at the 6-month follow-up in both groups. A further notable decrease was observed at 12 months compared to the 6-month time point, and this therapeutic effect was largely maintained over the subsequent 12-month treatment period. No significant intergroup differences in the number of MAs were found at the 6, 12, or 24-month follow-ups. However, at the 18-month assessment, the 5+PRN group exhibited a significantly higher number of MAs compared to the 5+T&E group (*P* < 0.05) (**Table 4**).

**Table 4:** Changes in the number of MAs before and after treatment.

MAs	Baseline	6m	12m	18m	24m	*F*	*P*
5+PRN(n=60)	89.74 ± 22.10	59.63 ± 17.88	44.56 ± 19.08	48.32 ± 19.25	46.34 ± 17.51	57.60	0.0000
5+T&E(n=59)	84.29 ± 21.20	60.98 ± 20.83	41.65 ± 17.73	41.03 ± 17.87	43.52 ± 18.40	55.49	0.0000
*t*	1.3707	0.3781	1.0608	2.1377	0.8565		
*P*	0.1731	0.7060	0.2909	0.0346	0.3934		

In the 5+PRN group, compared with baseline, the *q*-values were 12.1254, 18.1989, 16.6842, and 17.4795 at 6, 12, 18, and 24 months after treatment, respectively (all *P* < 0.01). In the 5+T&E group, the corresponding *q*-values were 9.2960, 17.0018, 17.2481, and 16.2562 at each follow-up time point (all *P* < 0.01). Pairwise comparisons among the five sample means were conducted using the Newman–Keuls *q*-test.

MA, microaneurysm;m, months.

### 3.5 Changes in retinal venular diameter before and after treatment

At baseline, no statistically significant difference in retinal venular diameter was observed between the 5+PRN group (143.43 ± 13.76 μm) and the 5+T&E group (144.05 ± 12.84 μm). In the 5+PRN group, a significant reduction in venular diameter was observed at 18 months (128.20 ± 16.00 μm) and 24 months (125.40 ± 13.87 μm) post-treatment compared with baseline (143.43 ± 13.76 μm) (*P* < 0.01). Similarly, the 5+T&E group exhibited a significant decrease at 18 months (127.48 ± 16.10 μm) and 24 months (123.20 ± 16.58 μm) relative to their baseline measurement (144.05 ± 12.84 μm) (*P* < 0.01). No statistically significant differences in venular diameter were found between the 5+PRN and 5+T&E groups at either the 18-month or 24-month follow-up assessments (**Table 5**).

**Table 5:** Changes in retinal venular diameter before and after treatment.

	Baseline	6m	12m	18m	24m	*F*	*P*
5+PRN(n=60)	143.43 ± 13.76	14.70 ± 14.73	141.78 ± 15.13	128.20 ± 16.00	125.40 ± 13.87	24.35	0.000
5+T&E(n=59)	144.05 ± 12.84	143.93 ± 16.18	142.25 ± 17.01	127.48 ± 16.10	123.20 ± 16.58	23.77	0.000
*t*	0.2579	0.6229	0.1573	0.2441	0.7842		
*P*	0.7969	0.5346	0.8753	0.8076	0.4345		

In the 5+PRN group, compared with baseline, the *q*-values were 8.0106 and 9.4861 at 18 and 24 months after treatment, respectively (all *P* < 0.01). In the 5+T&E group, the *q*-values were 8.0500 and 10.1300 at 18 and 24 months after treatment, respectively (all *P* < 0.01). Pairwise comparisons among the five sample means were conducted using the Newman–Keuls *q*-test.

m, months

### 3.6 Changes in DCP-VD before and after treatment

At baseline, no statistically significant difference in DCP-VD was observed between the 5+PRN group (56.23 ± 5.38%) and the 5+T&E group (56.59 ± 4.37%). In the 5+PRN group, DCP-VD showed a significant increase at 18 months (58.84 ± 5.20%) and 24 months (60.23 ± 5.03%) post-treatment compared with baseline (56.23 ± 5.38%) (*P* < 0.05 for both). Similarly, the 5+T&E group exhibited a significant improvement in DCP-VD at 18 months (59.25 ± 4.96%) and 24 months (60.16 ± 5.83%) relative to their baseline value (56.59 ± 4.37%) (*P* < 0.05 for both). No statistically significant differences in DCP-VD were found between the two groups at either the 18-month or 24-month follow-up assessments (**Table 6**).

**Table 6:** Changes in DCP-VD before and after treatment.

DCP-VD	Baseline	6m	12m	18m	24m	*F*	*P*
5+PRN(n=60)	56.23 ± 5.38	57.75 ± 5.76	57.30 ± 4.24	58.84 ± 5.20	60.23 ± 5.03	5.31	0.0004
5+T&E(n=59)	56.59 ± 4.37	57.99 ± 4.75	58.49 ± 5.53	59.25 ± 4.96	60.16 ± 5.83	4.06	0.0032
*t*	0.4044	0.2488	1.3200	0.4317	0.0760		
*P*	0.6867	0.8040	0.1894	0.6667	0.9396		

In the 5+PRN group, compared with baseline, the *q*-values were 3.9403 at 18 months after treatment (*P* < 0.05) and 6.0318 at 24 months after treatment (*P* < 0.01). In the 5+T&E group, the *q*-values were 3.9880 at 18 months after treatment (*P* < 0.05) and 5.3564 at 24 months after treatment (*P* < 0.01). Pairwise comparisons among the five sample means were conducted using the Newman–Keuls *q*-test.

DCP-VD, deep capillary plexus vascular density; m, months

### 3.7 Changes in the FAZ area before and after treatment

At baseline, no statistically significant difference in FAZ area was observed between the 5+PRN group (0.57 ± 0.09 mm²) and the 5+T&E group (0.58 ± 0.09 mm²). In the 5+PRN group, the FAZ area showed a gradual decrease to 0.56 ± 0.11 mm² at 18 months and 0.53 ± 0.10 mm² at 24 months post-treatment, compared to the baseline value of 0.57 ± 0.09 mm²; however, these changes were not statistically significant. Similarly, in the 5+T&E group, the FAZ area exhibited a gradual reduction to 0.55 ± 0.11 mm² at 18 months and 0.54 ± 0.09 mm² at 24 months relative to baseline (0.58 ± 0.09 mm²), but these differences also did not reach statistical significance. No statistically significant differences in FAZ area were found between the 5+PRN and 5+T&E groups at any post-treatment time point (6, 12, 18, or 24 months) (**Table 7**).

**Table 7:** Changes in the FAZ area before and after treatment.

FAZ(mm²)	Baseline	6m	12m	18m	24m	*F*	*P*
5+PRN(n=60)	0.57 ± 0.09	0.59 ± 0.10	0.57 ± 0.11	0.56 ± 0.11	0.53 ± 0.10	2.32	0.0574
5+T&E(n=59)	0.58 ± 0.09	0.58 ± 0.11	0.58 ± 0.10	0.55 ± 0.11	0.54 ± 0.09	2.21	0.0676
*t*	0.8116	0.4110	0.5156	0.1637	0.5035		
*P*	0.4187	0.6818	0.6071	0.8702	0.6156		

No statistically significant differences were observed at any post-treatment time point compared with baseline in either the 5+PRN group or the 5+T&E group. Pairwise comparisons among the five sample means were conducted using the Newman–Keuls *q*-test.

FAZ, foveal avascular zone; m, months

### 3.8 Changes in the HE area before and after treatment

In the 5+PRN group at the 24-month post-treatment assessment, the number of patients with an HE area of < 1.5 mm² increased from 4 to 17, while the number of patients with an HE area of 1.5–3.5 mm² decreased from 43 to 40, and those with an HE area of > 3.5 mm² decreased from 13 to 3. Similarly, in the 5+T&E group at 24 months, the number of patients with an HE area of < 1.5 mm² increased from 8 to 18, those with an HE area of 1.5–3.5 mm² decreased from 46 to 40, and those with an HE area > 3.5 mm² decreased from 5 to 1. These changes in the distribution of HE area from baseline to 24 months were statistically significant in both groups (*P* < 0.05) (**Table 8**).

**Table 8:** Changes in the HE area before and after treatment.

	HE area(mm^2^)	Baseline	24m	*χ^2^*	*P*
5+PRN(n=60)	< 1.5	4	17	14.4061	0.0007
	1.5–3.5	43	40		
	> 3.5	13	3		
5+T&E(n=59)	< 1.5	8	18	6.9314	0.0313
	1.5–3.5	46	40		
	> 3.5	5	1		
*χ^2^*		4.9820	1.0202		
*P*		0.0828	0.6004		

HE, hard exudate;m, months.

### 3.9 Changes in hemorrhage area before and after treatment

In the 5+PRN group at the 24-month post-treatment assessment, the number of patients with a hemorrhage area < 2 mm² increased from 8 to 16, those with an area of 2–4 mm² increase from 26 to 30, and those with an area > 4 mm² decreased from 26 to 14. Similarly, in the 5+T&E group at 24 months, patients with a hemorrhage area < 2 mm² increased from 5 to 18, those with an area of 2–4 mm² decreased from 36 to 30, and those with an area > 4 mm² decreased from 18 to 11. The changes in hemorrhage area distribution from baseline to 24 months were statistically significant in both groups (*P* < 0.05) (**Table 9**).

**Table 9:** Changes in hemorrhage area before and after treatment.

	hemorrhage area (mm^2^)	Baseline	24m	*χ^2^*	*P*
5+PRN(n=60)	< 2	8	16	6.5524	0.0378
	2–4	26	30		
	> 4	26	14		
5+T&E(n=59)	< 2	5	18	9.5829	0.0083
	2–4	36	30		
	> 4	18	11		
*χ^2^*		3.7516	0.4693		
*P*		0.1532	0.7909		

m, months.

### 3.10 Comparison of the number of injections and follow-up visits between the two groups

The mean number of injections within the 24-month follow-up was 14.02 ± 2.05 in the 5+PRN group and 11.44 ± 1.74 in the 5+T&E group. Meanwhile, the average number of follow-up visits was 24.89 ± 1.67 in the 5+PRN group and 13.94 ± 2.60 in the 5+T&E group. Both the injection frequency and follow-up visits were significantly lower in the 5+T&E group than in the 5+PRN group (all *P* < 0.01).

### 3.11 Number of patients receiving PRP in the two groups

A total of 6 patients in the 5+PRN group and 10 patients in the 5+T&E group received PRP. These patients were found to have retinal neovascularization at baseline FFA examination, and all PRP operations were performed after the completion of five consecutive monthly loading injections. No patients required PRP due to progressive diabetic retinopathy during subsequent treatment and follow-up.

### 3.12 Adverse effects

During the 24-month follow-up period, one patient developed mild subconjunctival hemorrhage after intravitreal injection, which resolved without special intervention. No adverse events such as endophthalmitis, persistently elevated intraocular pressure, or accelerated cataract progression were observed in either group.

## 4 Discussion

This 24-month retrospective comparative study evaluated the efficacy and injection frequency of conbercept administered via the 5+PRN and 5+T&E regimens in real-world patients with DME. The results demonstrated that the 5+T&E regimen achieved visual and anatomical outcomes comparable to those of the 5+PRN regimen while significantly reducing the injection burden. Therefore, for patients with DME, initial five loading injections followed by individualized maintenance treatment can maintain satisfactory therapeutic effects, reduce the total injection number, alleviate patients’ economic burden, and decrease overall medical costs.

### 4.1 5+T&E regimen for DME: a safe and effective treatment strategy

The efficacy and safety of intravitreal conbercept for DME have been established by the SAILING Study—a 12-month, multicenter, randomized, double-masked, double-dummy, parallel-controlled Phase III trial—and its subsequent 12-month extension study. The SAILING Study demonstrated that, compared with laser photocoagulation, intravitreal conbercept yielded a significantly greater improvement in BCVA from baseline at the 12-month endpoint. In the extension phase, patients initially assigned to the laser group were switched to conbercept treatment, which led to a notable improvement in BCVA; at the 24-month time point, BCVA in this crossover group was comparable to that in the original conbercept group (10). A real-world retrospective study showed that the 3+PRN regimen of conbercept combined with PRP in patients with high-risk PDR produced significant improvements in BCVA, CFT, retinal venular diameter, MA count, neovascularization area, and HE area relative to baseline over two years (11). Furthermore, the combination therapy group achieved better outcomes in terms of improvements in venular diameter, MA count, and HE area compared with the PRP-alone group. In addition, multiple clinical studies have confirmed that conbercept effectively improves BCVA and CFT in patients with DME. The present study further verified that both the 5+PRN and 5+T&E regimens of conbercept achieve satisfactory efficacy in improving BCVA and retinal anatomical parameters in DME patients.

A 2016 randomized controlled trial (RCT) enrolled 150 eyes from 116 subjects and randomized them at a 1:2:2 ratio into three groups: a monthly treatment group, a T&E group, and a T&E combined with laser group, with a 1-year follow-up. Patients in the monthly treatment group received intravitreal injections of 0.3 mg ranibizumab every four weeks. The T&E and T&E plus laser groups received monthly intravitreal 0.3 mg ranibizumab injections for the first four months, after which treatment and follow-up intervals were adjusted according to central retinal thickness (CRT), with the maximum treatment interval not exceeding 12 weeks. The results showed comparable improvements in BCVA and retinal anatomical outcomes among all three groups. However, the mean annual number of injections was significantly lower in the T&E and T&E plus laser groups compared with the monthly treatment group. This RCT demonstrated that the T&E regimen effectively improved BCVA and retinal structure while substantially reducing patient burden and healthcare resource utilization (12). In the second year of the study, the maximum treatment interval was extended to 16 weeks. Overall, 119 eyes (79%) completed the follow-up, and the visual and anatomical benefits achieved in the first year were well maintained (13). By the third year, 109 eyes (73%) remained in follow-up. The therapeutic improvements obtained over the first two years were sustained throughout the third year, and the mean number of injections decreased significantly in all groups (14). These findings indicate that the T&E regimen is a reasonable and effective long-term strategy for the management of DME. Nevertheless, the treatment extension criteria in this study relied solely on OCT-measured CRT. This strategy may overlook alterations in parafoveal retinal thickness that are associated with disease activity.

In a two-year RCT, patients with DME were randomly assigned in a 1:1:1 ratio to three groups: a T&E plus laser group, a T&E monotherapy group, and a PRN group (15). All patients in each group initially received monthly intravitreal injections of 0.5 mg ranibizumab until BCVA stabilization was achieved, defined as no change in BCVA over three consecutive monthly assessments. Once BCVA stability was confirmed, treatment was temporarily withheld. Subsequently, treatment intervals in the T&E groups were gradually extended: first to two months; if BCVA remained stable after the two-month interval, the interval was further extended to three months, serving as the maximum treatment interval. If BCVA decreased at any extended interval, treatment was resumed on a monthly basis. In the PRN group, after initial BCVA stabilization, patients were followed monthly, and treatment was reinitiated only upon documented BCVA loss. The results demonstrated that from baseline to month 12, the mean BCVA improved by 5.9, 6.1, and 6.2 letters in the T&E plus laser, T&E, and PRN groups, respectively. By month 24, the improvements were 8.3, 6.5, and 8.1 letters, respectively. The T&E regimens were non-inferior to the PRN regimen in terms of efficacy. Moreover, beyond initial BCVA stabilization through month 24, over 70% of patients in the T&E groups maintained BCVA stability with treatment intervals of at least two months. The mean number of injections over 24 months was 12.4 in the T&E plus laser group, 12.8 in the T&E group, and 10.7 in the PRN group. The slightly higher number of injections in the T&E groups compared with the PRN group was attributed to a more conservative initial dosing strategy. This conservative approach was adopted by clinicians during early clinical application of the T&E protocol to avoid potential vision loss related to interval extension. From initial BCVA stabilization to month 24, the mean numbers of follow-up visits were 9.0, 8.9, and 16.6 in the T&E plus laser, T&E, and PRN groups, respectively. This represented a 46% reduction in follow-up visits with the T&E strategy compared with the PRN approach. The study indicates that the T&E regimen can serve as a viable alternative to the PRN regimen for managing DME. The PRN approach requires monthly follow-ups with uncertain treatment decisions at each visit, which may lead to poorer patient compliance. In contrast, the T&E strategy adopts an individualized and proactive schedule with gradual interval extension, thereby reducing clinic visits, lowering treatment burden, and potentially improving long-term treatment adherence.

The VIOLET study also confirmed that the T&E regimen can reduce both the number of injections and follow-up visits for patients with DME. This RCT enrolled 500 patients and randomly assigned them in a 1:1:1 ratio to a T&E group, a PRN group, and a fixed-dosing group (16). The results demonstrated that the changes in BCVA from baseline at both week 52 and week 100 in the T&E and PRN groups were non-inferior to those achieved with the fixed-dosing regimen. There were no statistically significant differences in CRT improvement among the three groups. Furthermore, the T&E group had the fewest injections and follow-up visits among the three treatment strategies, which was consistent with the findings from our study.

### 4.2 Anti-VEGF therapy for improvement of DME and regression of DR

In the present study, we found that both the 5+PRN and 5+T&E conbercept regimens significantly improved BCVA, reduced CFT, decreased the number of MAs, narrowed retinal venular diameter, increased vessel density in the DCP-VD, and reduced the areas of HE and hemorrhages in DME patients. These findings are consistent with those of numerous previous studies.

#### 4.2.1 Improvement in BCVA and CFT

DME is the leading cause of vision impairment in patients with DR, and OCT is widely used in its diagnosis and evaluation. In 1999, Otani et al. conducted an OCT-based comparative study of 59 eyes from 42 DME patients and 10 eyes from normal controls to assess the correlation between retinal thickness at the fovea and BCVA (17). The results indicated that DME manifestations on OCT could be categorized into three primary structural alterations: diffuse retinal thickening (DRT), cystoid macular edema (CME), and serous retinal detachment (SRD), with some eyes exhibiting more than one type. DRT was the most frequent manifestation, observed in 52 of the 59 eyes (88%). On OCT, an edematous retina exhibited a sponge-like appearance with significant thickening and expanded hyporeflective areas, typically in the outer retinal layers. CME was identified in 28 eyes (47%), with cysts predominantly located in the outer retina; in severe cases, large cysts could involve nearly the full retinal thickness. SRD was present in 9 eyes (15%), appearing as a hyporeflective space between the neurosensory retina and the retinal pigment epithelium. Related studies suggest that DRT and CME are mainly caused by the breakdown of the i-BRB, as well as cytoplasmic swelling and necrosis of Müller cells, whereas SRD results from dysfunction of the e-BRB, causing subretinal fluid accumulation. Furthermore, Otani’s study concluded that BCVA was correlated solely with retinal thickness, independent of the specific OCT morphological features.

Our findings are consistent with multiple prospective and retrospective studies demonstrating that anti-VEGF therapy leads to a significant reduction in retinal thickness and improvement in BCVA in most patients with DME. However, some studies have reported that over 30% of patients still exhibit persistent DME despite intensive anti-VEGF treatment, suggesting the potential involvement of chronic inflammation. For such patients, intravitreal corticosteroid injection or PPV may represent more effective therapeutic options. OCT can be utilized to identify biomarkers associated with a poor response to anti-VEGF therapy, thereby facilitating early prediction of treatment response and guiding clinical decision-making.

OCT enables the evaluation of intraretinal cystoid fluid—including its location, size, and reflectivity—to assess prognosis and elucidate pathogenesis in DME. Cysts located in the outer nuclear layer (ONL) and outer plexiform layer (OPL) may be associated with microaneurysms and vascular leakage originating from the DCP. Cysts in the inner nuclear layer (INL) are often linked to leakage from the SCP and disruption of the i-BRB. Cystoid fluid near the ganglion cell layer may indicate more severe DME and correlate with prolonged visual recovery and a higher number of anti-VEGF injections, as the tightly packed GCL is less accommodating to fluid accumulation (18). Large, confluent intraretinal cysts often suggest chronicity and greater disease severity, potentially reflecting impaired fluid clearance due to Müller cell dysfunction or necrosis. Such cysts may also indicate concurrent damage to both the inner and outer BRB, accompanied by significant macular ischemia (19). Disorganization of the retinal inner layers (DRIL), defined as the inability to distinguish boundaries between the ganglion-INL complex and INL-OPL, is a predictive biomarker for visual impairment and poor treatment response in DME. DRIL suggests disorganization or loss of inner retinal neurons, disrupting signal transmission from photoreceptors to ganglion cells and thereby impairing vision. DRIL involving the foveal center has the most profound impact on visual acuity; studies report that every 100-μm increase in DRIL is associated with a loss of 4.7 letters in visual acuity (20). Early resolution of DRIL following anti-VEGF therapy may indicate a favorable visual prognosis. Furthermore, DRIL is strongly associated with epiretinal membrane presence, as well as disruption of the external limiting membrane (ELM) and ellipsoid zone (EZ). This suggests that the pathological processes underlying DRIL may also damage outer retinal structures. Disorganization of the retinal outer layers (DROL) is characterized by disruption of the ELM, EZ, interdigitation zone (IZ), and RPE on OCT. The presence of DROL in DME suggests photoreceptor damage and may be related to macular ischemia (21).

#### 4.2.2 Reduction in MA count

MAs form as a result of endothelial cell proliferation, basement membrane thickening, and pericyte loss induced by chronic hyperglycemia, and typically represent the earliest clinical sign of DR. On fundus examination, MAs appear as tiny red dots scattered throughout the retina, serving as a hallmark feature of DR and DME, as well as a key criterion for the clinical diagnosis of DR. FFA remains the gold standard for detecting MAs, which present as hyperfluorescent spots in the early phases. On OCT, MAs are visualized as round or oval, well-defined hyperreflective lesions. In DR, MAs are often located adjacent to non-perfused areas or alongside dropped-out capillaries, indicating retinal ischemia. In DME, a higher density of MAs is observed in the peri-edematous region compared with the central edematous area. Therefore, we propose that active leakage from MAs in the peri-edematous zone may contribute to the expansion of retinal edema (22).

Studies have shown that MAs do not remain stable over time in the retinas of patients with DR or DME. MA turnover (MAT), which encompasses the MA formation rate and disappearance rate, generally reflects disease activity and serves as an important predictor of DME progression. Eyes with low MAT exhibit less disease activity and a lower risk of deterioration. Previous research has demonstrated that following anti-VEGF therapy, MAs in DME patients tend to shrink and disappear, while the formation of new MAs is suppressed. The number of MAs that disappear exceeds the number of newly formed ones, leading to a net reduction in the total MA count. This suggests that MAT may serve as a biomarker for treatment response in patients with DR and DME (23). Studies have revealed that DME patients with a poor response to anti-VEGF therapy typically exhibit more extensive damage and a higher density of MAs in the DCP compared with those with a favorable response (24). Another study further demonstrated that the number of MAs in areas with persistent edema following anti-VEGF treatment was significantly greater than that in regions where edema had resolved (25). Based on these findings, we hypothesize that a high MA density serves as both a risk factor for residual edema after anti-VEGF therapy and a prognostic indicator of suboptimal treatment response in DME.

This study further supports the aforementioned perspective, demonstrating a significant reduction in the number of MAs in both the 5+PRN and 5+T&E conbercept treatment groups by the 6-month follow-up time point. In this research, FFA was employed for the quantification of MAs. The primary advantage of FFA lies in its ability to clearly delineate the location and morphology of MAs, facilitating accurate counting and allowing for the assessment of leakage activity. However, FFA has notable limitations. Firstly, it is an invasive procedure. Moreover, FFA cannot differentiate between retinal and choroidal vascular structures, thereby limiting precise determination of the vascular layer in which MAs reside. OCTA enables layer-specific visualization of MAs by distinguishing between the superficial capillary plexus, deep capillary plexus, and choroidal vasculature. However, its detection rate for MAs is lower than that of FFA. A study by Schreur et al. reported that only approximately 58% of MAs identified by FFA could be detected using OCTA (26). This discrepancy may be attributed to two main factors: firstly, OCTA may fail to visualize MAs located within capillary saccules with slow or absent blood flow due to diminished signal intensity; secondly, not all hyperfluorescent spots observed on FFA represent true MAs, as some may arise from capillary wall staining or leakage of contrast agent from damaged vessels, mimicking the appearance of MAs.

#### 4.2.3 Reduction in retinal venular diameter

Retinal venular dilation is a significant marker in the progression of DR, and the “beading” appearance of retinal veins serves as one of the grading criteria in DR staging systems. A retrospective cross-sectional study reported that venous beading was present in 41.3% (327/791) of eyes with PDR, 5.9% (31/526) of eyes with severe NPDR, and 0% (0/295) of eyes with moderate NPDR. Among the 225 eyes exhibiting venous beading in two or more quadrants, 214 (95.1%) were graded as PDR. The study also identified that the formation of venous beading was significantly associated with capillary non-perfusion, duration of diabetes, and smoking history, and represented an independent risk factor for PDR (27).

Under hyperglycemic conditions, the initiation of inflammatory and oxidative stress responses in the retina leads to abnormal leukocyte adhesion and accumulation of advanced glycation end products (AGEs) within retinal vessels. These processes contribute to retinal ischemia, hypoxia, and damage to vascular endothelial cells. Concurrently, patients with DR exhibit morphological alterations in retinal microvessels, including basement membrane thickening, loss of tight junctions between endothelial cells, and pericyte loss. These changes exacerbate hypoxia and weaken the vascular wall structure. Chronic stimulation by prolonged hypoxia and inflammation induces compensatory dilation of retinal veins, followed by the development of venous beading (28). Studies have indicated that an increase in retinal venular diameter is associated with DR progression, with larger diameters correlating with more advanced DR stages, including a higher prevalence of PDR (29). Furthermore, wider retinal venular diameters have been confirmed as an independent risk factor for both the onset and progression of DR (30). Consequently, quantitative measurement of retinal vascular diameter provides additional clinical predictive information for assessing the risk of DR progression.

A 12-month prospective clinical study demonstrated that monthly aflibercept treatment for DME resulted in a significant reduction in retinal venular diameter after 3 months of therapy. This reduction was maintained over the subsequent 9 months, regardless of whether PRN treatment was administered (31). In our previously published clinical study investigating conbercept combined with PRP for DME, a non-significant decrease in retinal venular diameter was observed at the 6-month follow-up. However, a continuous downward trend was noted at 12, 18, and 24 months, with statistically significant differences compared with baseline values (11). A secondary analysis of the CLARITY study reported that while intraretinal hemorrhage and IRMA showed significant improvement after 52 weeks of aflibercept treatment for DME, no regression of venous beading was observed (32). The discrepancy between the CLARITY study findings and those of the present study may be attributed to differences in the observation period. Our study evaluated outcomes over 24 months, whereas venular diameter demonstrated statistically significant improvement only after 18 months of treatment. This suggests that venular remodeling requires a longer duration compared to functional and anatomical parameters such as BCVA, CRT, and MAs.

#### 4.2.4 Increase in DCP-VD

OCTA enables the identification of early microvascular alterations in patients with DR. Vessel density (VD) is defined as the proportion of the total measured area occupied by blood vessels in OCTA images. In DR, VD in both the SCP and DCP decreases due to capillary occlusion or dropout. A reduction in DCP-VD has been closely associated with photoreceptor cell damage. Studies have revealed that microvascular abnormalities, manifested as decreased VD in both the SCP and DCP on OCTA, can be detected in the early stages of DR or even in the pre-clinical phase. As DR severity progresses, OCTA can visualize extensive non-perfusion areas, indicating a worsening of retinal ischemia (33).

The size of the FAZ, whether measured by OCTA or FFA, shows a negative correlation with visual acuity (34). Studies have indicated that FAZ size is positively correlated with the overall severity and progression of DR (35). In patients with DR, FAZ size can serve as an indicator of DME severity; an enlarged FAZ area suggests DR progression and signifies more severe macular ischemia (36). Furthermore, studies have demonstrated that the FAZ morphology exhibits greater irregularity in patients with severe DME compared to those with early-stage DME (37).

In this study, no statistically significant reduction in the FAZ area was observed in either treatment group following anti-VEGF therapy. We propose several potential explanations for this finding. First, the 2-year observation period may have been insufficient to detect significant FAZ remodeling, as microvascular repair in DR patients is often a protracted process. Second, the wide physiological variability of the FAZ area in the general population may limit its sensitivity for evaluating treatment response (38). Furthermore, FAZ size exhibits inherent fluctuations over time; studies have reported changes in FAZ measurements between clinical visits regardless of therapeutic intervention (39). Additionally, coexisting epiretinal membranes or DME involving the foveal center can alter FAZ morphology, further complicating its interpretation. Therefore, relying solely on the FAZ area as an indicator of DR progression or regression in clinical practice may have limitations (38).

The FAZ circularity index (4πA/P²), where A represents the FAZ area, and P denotes the FAZ perimeter, can serve as an alternative metric for FAZ assessment. Under physiological conditions, the FAZ typically exhibits a near-circular shape surrounded by a continuous capillary ring. In DR, however, the loss of pericapillary vessels around the FAZ margin leads to significant contour alterations, resulting in an irregular and tortuous FAZ morphology. These changes reduce the circularity index without necessarily causing substantial alterations in the total FAZ area (40). Previous studies have demonstrated that the circularity index is a more sensitive indicator than area for detecting microvascular dropout in the perifoveal region (41). Therefore, the FAZ circularity index may be considered a valuable parameter for future clinical investigations.

### 4.3 Strengths and limitations of the study

This study is the first to compare the 24-month BCVA and retinal anatomical changes in DME patients treated with conbercept using either the 5+PRN or 5+T&E regimens, while also comparing the number of injections between the two regimens. It confirms that the 5+T&E regimen can reduce injection frequency while maintaining therapeutic efficacy, providing important evidence for clinical decision-making in DME management. However, several limitations should be considered. As a single-center retrospective study with a limited sample size, the findings require further validation through larger multicenter, prospective, randomized controlled trials. The measurements of retinal venular diameter, MA count, and areas of HE and hemorrhage were performed manually due to the lack of reliable automated analysis software, introducing potential measurement subjectivity. Finally, although no statistically significant change in FAZ area was observed on OCTA following anti-VEGF treatment, future studies should incorporate additional FAZ parameters to better evaluate microvascular improvements. This study also failed to record the baseline DR staging of enrolled patients. Heterogeneity in the baseline severity of DR may serve as a potential confounding factor affecting treatment outcomes. Further studies should therefore document DR staging at baseline to minimize such bias.

## 5 Conclusion

In conclusion, conbercept is a safe and effective treatment for DME. Both the 5+PRN and 5+T&E regimens significantly improved BCVA and retinal anatomical structure over the 24-month treatment period, with no statistically significant differences observed between the two groups. In both groups, improvements in BCVA, CFT, and MA count were observed early in the treatment course, while retinal venular diameter and vessel density in the DCP-VD showed significant improvement after 18 months of treatment. These findings suggest that conbercept can reverse vascular abnormalities in DME patients, although this process requires a longer duration. The 5+T&E regimen required fewer injections over the two-year period compared to the 5+PRN regimen. An individualized treatment approach can therefore ensure therapeutic efficacy while reducing the treatment burden on patients and healthcare systems.

## Data Availability

The raw data supporting the conclusions of this article will be made available by the authors, without undue reservation.
